# The relationship between lactate dehydrogenase and Apolipoprotein A1 levels in patients with severe pneumonia

**DOI:** 10.5937/jomb0-45782

**Published:** 2024-04-23

**Authors:** Jiang Wang, Ronghua Wang, Ying Zhou, Yao Ma, Chunyan Xiong

**Affiliations:** 1 Zhaotong First People's Hospital, Pulmonary and Critical Care Medicine, Zhaotong, China

**Keywords:** lactate dehydrogenase, ApoA1 level, patients, hospitalization, prognosis, value, aktat dehidrogenaza, nivoi ApoA1, pacijenti, hospitalizacija, prognoza, vrednost

## Abstract

**Background:**

To investigate the relationship between lactate dehydrogenase and apolipoprotein A1 levels and the condition and prognosis of patients with severe pneumonia.

**Methods:**

Data was collected from 204 patients with severe pneumonia who were hospitalized from January 1, 2019 to December 1, 2021 in Zhaotong First People's Hospital (respiratory intensive care unit (RICU)), and divided into survival group (160 patients) and death group (44 patients) according to their hospitalization outcome. The relationship between lactate dehydrogenase and apolipoprotein A1 levels and general information, disease, and treatment needs of patients with severe pneumonia was analyzed, and lactate dehydrogenase, apolipoprotein A1, neutrophil-to-lymphocyte ratio, hematocrit, C-reactive protein, calcitoninogen, D-dimer, Acute Physiology and Chronic Health Status Rating System II, and Pneumonia Severity Index scores were compared between the survival and death groups. The value of these indicators in determining the prognosis of patients was analyzed using subject operating characteristic (ROC) curves. Logistic regression was used to analyze the risk factors for death from severe pneumonia.

## Abstract (sr)

**Uvod:**

Cilj je bio da se istraži veza između nivoa laktat dehidrogenaze i apolipoproteina A1 i stanja i prognoze pacijenata sa teškom pneumonijom.

**Metode:**

Podaci su prikupljeni od 204 pacijenta sa teškom upalom pluća koji su bili hospitalizovani od 1. januara 2019. do 1. decembra 2021. godine u Prvoj Narodnoj bolnici Zhaotong (jedinica intenzivne nege respiratornih organa (RICU)), i podeljeni u grupu za preživljavanje (160 pacijenata) i smrt grupa (44 bolesnika) prema ishodu hospitalizacije. Analiziran je odnos između nivoa laktat dehidrogenaze i apolipoproteina A1 i opštih informacija, bolesti i potreba za lečenjem pacijenata sa teškom pneumonijom, a analizirana je i laktat dehidrogenaza, apolipoprotein A1, odnos neutrofila i limfocita, hematokrit, C-reaktivni protein, D kalcitoninogen. D-dimer, sistem ocenjivanja akutne fiziologije i hroničnog zdravstvenog statusa II, i indeks ozbiljnosti pneumonije I upoređeni su između grupa preživljavanja i smrti. Vrednost ovih pokazatelja u određivanju prognoze bolesnika analizirana je korišćenjem krivih operativnih karakteristika subjekta (ROC). Logistićka regresija je korišćena za analizu faktora rizika za smrt od teške pneumonije.

**Rezultati:**

Starost i tip pneumonije su se značajno razlikovali između dve grupe (P<0,05). Nije bilo značajnih razlika u polu i ukupnom broju dana hospitalizacije (P>0,05). Nivoi LDH (laktat dehidrogenaze) i ApoA1 (apolipoproteina A1) pokazali su značajne razlike među različitim starosnim grupama sa teškom pneumonijom (P<0,05). Nivoi LDH i ApoA1 nisu se značajno razlikovali između pacijenata sa SCAP (teška pneumonija stečena u zajednici) i SHAP (bolnički stečena pneumonija) (P>0,05). Nivoi LDH i ApoA1 bili su viši kod pacijenata sa teškom pneumonijom sa akutnom egzacerbacijom ili MODS-om (sindrom višeorganske disfunkcije), u poređenju sa onima bez (P<0,05). Nivoi LDH i ApoA1 značajno su varirali sa PSI (indeks ozbiljnosti pneumonije) ocenama ili APACHE II (akutna fiziologija i hronična zdravstvena evaluacija II) skor, trajanje boravka u intenzivnoj nezi i trajanje mehaničke ventilacije kod pacijenata sa teškom pneumonijom (P<0,05). Nivoi LDH i ApoA1 su bili značajno viši u grupi umrlih u poređenju sa grupom koja je preživela (P<0,05). Odnos neutrofila i limfocita (NLR), hematokrit (HCT), C-reaktivni protein (CRP), kalcitoninogen (PCT), D-dimer (DD), PSI rezultati i APACHE II rezultati nisu pokazali značajne razlike između ova dva grupe (P>0,05). LDH i ApoA1, kada su kombinovani, imali su veću prediktivnu vrednost za smrtnost od teške pneumonije (područje ispod krive, AUC=0,873, P<0,05). Multivarijantna logistička regresiona analiza je potvrdila da LDH>289 U/mL i ApoA1<0,92 mg/mL povećavaju rizik od teške smrtnosti od pneumonije (OR=4,275, 0,548, P<0,05).

**Zaključak:**

Povišeni nivoi LDH i smanjeni nivoi ApoA1 kod pacijenata sa teškom pneumonijom su vredni u proceni stanja i prognoze pacijenata i mogu pružiti pomoć u ranoj proceni stanja pacijenata i dijagnozi i lečenju.

## Introduction

Severe pneumonia, also known as toxic pneumonia or fulminant pneumonia, is pneumonia caused by various pathogens that cause substantial inflammation of the lung with severe infectious toxic symptoms and associated complications [1]. Severe pneumonia is a common disease in the respiratory intensive care unit (RICU), which is characterized by rapid onset, rapid progression, severe disease, and poor prognosis. In addition to the respiratory system, it often involves multiple organs throughout the body and is prone to respiratory failure, infectious shock, and even acute respiratory distress syndrome (ARDS) or multiple organ dysfunction syndrome (MODS), which are acute and critical respiratory system diseases [2] [3]. Although modern medical technology has made significant progress, the morbidity and mortality rate of severe pneumonia remains high. Therefore, it is crucial to actively search for practical, rapid and sensitive monitoring indicators and methods to evaluate the prognosis of severe pneumonia in a timely manner, adjust the treatment measures as early as possible and reduce the morbidity and mortality rate.

Lactate dehydrogenase (LDH) is an enzyme that exists in many tissues and cells and plays an important role in body metabolism. LDH is mainly involved in the metabolism of lactic acid and produces energy by catalyzing the conversion of lactic acid to pyruvate. Under normal conditions, LDH levels are low. However, under conditions such as inflammation and cell damage, lactate dehydrogenase is released into body fluids, resulting in elevated concentrations of LDH. Therefore, LDH levels can be used as an indicator of the degree of inflammation and cell damage. Lactate dehydrogenase (LDH) is an important indicator of myocardial injury and SP has a significant effect on myocardial function, triggering changes in the expression of inflammatory factors [4]. LDH is an important enzyme index, which is widely used to evaluate the degree of cell damage. Recent studies have shown that lactate dehydrogenase levels are closely related to the onset and prognosis of severe pneumonia. Severe pneumonia has a significant effect on myocardial function, resulting in elevated lactate dehydrogenase levels. Therefore, the monitoring of LDH can be used as one of the indicators of the degree of myocardial damage in patients with severe pneumonia. Apolipoprotein A1 (ApoA1) is a lipoprotein synthesized mainly by the liver and has important biological activity in plasma. ApoA1 is the main component of high-density lipoprotein (HDL), its functions include promoting cholesterol reflux, antioxidant, anti-inflammatory and so on. In addition, ApoA1 is involved in regulating the immune system and inflammatory response. Studies have found that ApoA1 levels are associated with cardiovascular disease risk, inflammatory status, and prognosis for certain infectious diseases. Apolipoprotein A1 (ApoA1) is an important inflammatory factor that responds to the degree of inflammatory response of the organism [5]. Apolipoprotein A1 (ApoA1) is a protein that is closely related to the inflammatory response. The study found that ApoA1 levels may be associated with the onset and severity of severe pneumonia. Severe pneumonia triggers an inflammatory response, leading to changes in ApoA1 expression levels. Quantitative analysis of ApoA1 can provide an assessment of the inflammatory response in patients with severe pneumonia.

However, the relationship between LDH and apolipoprotein A1 levels in patients with severe pneumonia remains unclear. Therefore, this study aimed to investigate the potential value of LDH and apolipoprotein A1 levels in diagnosis, assessment of disease severity, and prediction of prognosis in patients with severe pneumonia. By collecting and analyzing clinical data and laboratory test results from patients with severe pneumonia, we hope to be able to provide a more complete understanding and provide new guidance for the diagnosis and treatment of severe pneumonia.

## Materials and methods

### General information

A total of 204 patients with SP (Streptococcus pneumoniae) admitted to Zhaotong First People's Hospital from January 2019 to December 2021 were selected. The study complied with the relevant regulations of medical ethics and was approved by the Medical Ethics Committee of Zhaotong First People's Hospital, and patients and their families gave their informed consent.

### Incorporation criteria

(1) The diagnostic criteria for community-acquired pneumonia or hospital-acquired pneumonia/ventilator-associated pneumonia need to be met firstly [6] and secondly for severe pneumonia [7]; (2) Complete clinical information; (3) Not receiving treatment prior to admission.

### Exclusionary criteria

(1) Combined malignancy, multiple myeloma, burns, Cushing's syndrome, primary or secondary hyperthyroidism, primary or secondary hypothyroidism, mania, myasthenia gravis, malignant dystrophy, acute major blood loss disorders, etc. Because the above diseases have a greater impact on the catabolism of the body, they are excluded in order to reduce the influence of confounding factors; (2) Chronic kidney disease stage 3 or greater (estimated glomerular filtration rate <30 mL/ (min×1.73 m^2^) or admission serum creatinine >354 μmol/L, acute urinary tract obstruction, bladder rupture; (3) Cirrhosis, subacute liver failure and slow plus acute (subacute) liver failure of intermediate and advanced stages, admission serum total bilirubin > 10 times the upper limit of normal; (4) Receiving renal replacement therapy prior to and during this hospitalization; (5) Missing data: missing parameters required for the first admission renal function, blood count, serum inflammatory index, Acute Physiology and Chronic Health Status Rating System II (APACHE II) score and Pneu -monia Severity Index (PSI) score; (6) Pregnant women. For repeat patients with multiple hospitalizations during the study period, the last hospitalization data was selected as the baseline indicator. Approval consent for this study was obtained from the hospital ethics committee.

### Grouping and data collection

The primary outcome of this study was all-cause mortality during hospitalization. The group was divided into survival and death groups according to the outcome of this hospitalization. Access to enrolled patients through electronic medical record system, (1) General information: gender, age, pneumonia typing, length of stay in RICU, total length of stay, etc; (2) Related complications: history of surgery during hospitalization, acute hepatic and renal insufficiency, ARDS, respiratory failure, acute exacerbation of chronic obstructive pulmonary disease (COPD), hypo -proteinemia, sepsis, shock, MODS, etc; (3) Inpatient treatment measures: use of sedation and analgesia, tracheal intubation, length of mechanical ventilation, etc; (4) Laboratory test results (collected for the first time after admission): LDH, ApoA1, neutrophil-to-lymphocyte ratio (NLR), hematocrit (HCT), C-reactive protein (CRP), calcitoninogen (PCT), D-dimer (DD), etc; The activity of LDH was measured by photometry. The concentration of ApoA1 is determined by immunoassay by binding reaction with ApoA1 specific antibody. HCT and NLR are measured using an automated blood cell analyzer. The concentrations of CRP and PCT were determined by immunofluorescence. ELISA is used to determine the concentration of DD by detecting the signal generated by the binding of specific antibodies to DD; (5) Disease severity score: Acute Physiology and Chronic Health Status Rating System II (APACHE II) score and Pneumonia Severity Index (PSI) score on the day of admission. This study promises to keep all patient data strictly confidential and not for any commercial use.

### Clinical observation index

(1) To compare the differences in general information, lactate dehydrogenase, ApoA1, NLR, CRP, PCT, DD, APACHE II score, and PSI score levels between patients in the survival and death groups; (2) Analysis of the relationship between general data, complications, inpatient treatment measures, and disease severity scores and LDH and ApoA1 levels; (3) Comparison of the prognostic value of each index of LDH, ApoA1, NLR, CRP, PCT, DD, APACHE II score, and PSI score; (4) To analyze the factors affecting the prognosis of patients with severe pneumonia.

### Statistical analysis

Statistic Package for Social Science (SPSS) 21.0 was used for analysis, and the measurement data that conformed to normal distribution in the experimental data were expressed by x̄±s, and the comparison of two groups was done by the paired t-test, and thecount data were expressed as cases or rates, and the comparison of two groups was done by the χ^2^ test, and those that were statistically significant in the single factor analysis were included in the multifactor analysis, and the logistic regression model was used for the multifactor analysis, and the receiver operating characteristic’ curve (ROC ) curve was used to evaluate the predictive value of related factors, and receiver operating characteristic’ curve (ROC) curve was used to evaluate the predictive value of lactate dehydrogenase and apolipoprotein A1 levels on the poor prognosis of SMPP (Severe Mycoplasma pneumoniae pneumonia), and the difference was considered statistically significant at *P* < 0.05.

## Results

General information

When comparing age and pneumonia typing between the two groups, the difference was statistically significant (*P*<0.05). There was no statistically significant difference between the two groups when comparing gender and total hospital stay (*P*>0.05). (Table 1).

**Table 1:** Comparison of general information between the two groups (cases (%)/x̄±s).

Index	Death group<br>(n=44)	Survival<br>group<br>(n=160)	t/χ^2^	p
Age	80.54±8.15	75.12±9.43	3.471	0.000
Gender				
Male	28	98	0.083	0.773
Female	16	62		
Pneumonia typing				
SCAP	32	138	4.544	0.033
SHAP	12	22		
Total hospitalization time (d)	17.34±5.97	18.08±5.04	0.828	0.409

### Relationship between demographic information and admission LDH and ApoA1

There was no statistically significant difference in LDH and ApoA1 levels between male patients and female patients (*P*>0.05). The differences in LDH and ApoA1 levels were statistically significant when comparing patients with severe pneumonia at different ages (*P*<0.05) (Table 2).

**Table 2:** Relationship between demographic information and admission LDH and ApoA1.

Index	Groups	Cases	LDH	ApoA1
Gender	Male	126	274.32±29.12	0.89±0.27
	Female	78	270.12±30.19	0.93±0.25
t			0.987	1.057
P			0.325	0.292
Age	≤70 years old	61	236.28±27.14	1.08±0.34
	>70 years old	143	293.14±31.02	0.81±0.25
t			12.427	6.311
P			0.000	0.000

### The relationship between severe pneumonia disease and admission LDH and ApoA1

The difference in LDH and ApoA1 levels between SCAP (Severe Community-Acquired Pneumonia) and SHAP (hospital-acquired pneumonia) patients was not statistically significant (*P*>0.05). LDH and ApoA1 levels in patients with severe pneumonia who had acute exacerbation of chronic obstructive pulmonary disease or MODS combined during hospitalization were higher than those in patients with severe pneumonia without acute exacerbation of chronic obstructive pulmonary disease or MODS, and the difference was statistically significant (*P*<0.05). The differences in LDH and ApoA1 levels were statistically significant (*P*<0.05) when comparing patients with severe pneumonia with different PSI grades or APACHE II scores. The differences in LDH and ApoA1 levels were statistically significant (*P*<0.05) when comparing patients with severe pneumonia with different ICU length of stay (Table 3).

**Table 3:** The relationship between severe pneumonia disease and admission LDH and ApoA1.

Index	Groups	Cases	LDH	ApoA1
Pneumonia typing	SCAP	170	275.45±29.23	0.88±0.26
	SHAP	34	271.12±30.32	0.92±0.25
t			0.784	0.824
P			0.434	0.411
Acute exacerbation of chronic<br>obstructive pulmonary disease	No	160	241.16±27.38	1.12±0.36
	Yes	44	291.43±31.18	0.88±0.29
t			10.460	4.071
P			0.000	0.000
MODS	No	140	253.27±28.20	1.16±0.37
	Yes	64	278.29±30.35	0.89±0.22
t			5.740	5.412
P			0.000	0.000
RICU length of hospitalization	≤ 10 d	123	247.07±27.21	1.18±0.31
	>10 d	81	284.27±30.54	0.86±0.28
t			9.098	7.492
P			0.000	0.000
PSI Score	≤ 3 level	60	238.69±27.25	1.12±0.34
	>3 level	144	291.15±31.12	0.76±0.26
t			11.365	8.201
P			0.000	0.000
APACHE Score	≤ 19 point	112	239.15±27.76	1.22±0.35
	>19 point	82	287.29±31.16	0.71±0.20
t			11.327	11.849
P			0.000	0.000

### Relationship between treatment need and admission LDH and ApoA1

There was no statistically significant difference in LDH and ApoA1 levels when comparing patients with severe pneumonia who required tracheal intubation or sedation and analgesia during hospitalization (*P*>0.05). LDH and ApoA1 levels in patients with severe pneumonia with different duration of mechanical ventilation were compared with statistically significant differences (*P*<0.05) (Table 4).

**Table 4:** Relationship between treatment need and admission LDH and ApoA1.

Index	Groups	Cases	LDH	ApoA1
Sedation and analgesia	No	150	274.47±29.28	0.89±0.27
	Yes	54	271.27±30.31	0.91±0.26
t			0.682	0.471
P			0.496	0.638
Tracheal intubation	No	126	248.37±27.48	1.17±0.31
	Yes	78	286.42±30.22	0.81±0.24
t			9.249	8.757
P			0.000	0.000
Duration of the mechanical period	≤ 180 h	102	252.26±28.27	1.16±0.32
	>180 h	102	273.27±30.30	0.89±0.23
t			5.120	6.920
P			0.000	0.000

### Relationship between indicators and prognosis

LDH and ApoA1 levels in the death group were 105.08 (75.22~140.0), which was significantly higher than 86.66 (62.66~106.14) in the survival group, with statistically significant differences (*P*<0.05). There was no statistically significant difference in the levels of NLR, HCT, CRP, PCT, DD, PSI scores, and APACHE II scores between the two groups (*P*>0.05) (Table 5).

**Table 5:** Comparison of prognostic-related indicators between the two groups (x̄±S).

Index	Death group (n=44)	Survival group (n=160)	t	P
LDH (U/mL)	319.32±38.21	225.76±26.98	18.488	0.000
ApoA1 (mg/mL)	0.85±0.26	1.02±0.32	3.240	0.001
NLR	9.38±2.09	9.76±3.02	0.784	0.434
HCT (%)	33.65±8.23	34.65±8.19	0.717	0.474
CRP (mg/L)	58.89±17.98	60.57±18.95	0.526	0.599
PCT (ng/mL)	0.47±0.11	0.44±0.10	1.724	0.086
DD (mg/L)	2.38±0.37	2.29±0.48	1.152	0.250
PSI Score	126.34±35.58	122.38±30.28	0.739	0.461
APACHEII Score	19.17±3.98	19.35±4.09	0.260	0.795

### Comparison of prognostic values

**Figure 1:**
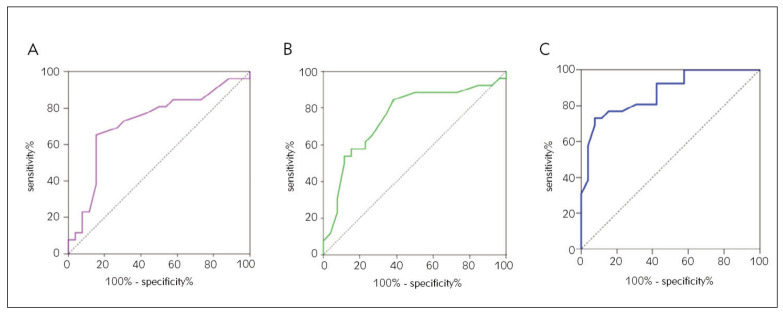
ROC curves of LDH (A), ApoA1 (B), and LDH combined with ApoA1 (C) to predict prognosis of patients with severe pneumonia.

The AUC (Area Under the Curve) of LDH for predicting death in patients with severe pneumonia was 0.723 (95% CI (0.579~0.868)), with a sensitivity of 70.7% and specificity of 71.8% at a cut-off value of 289 U/mL. ApoA1 predicted death in patients with severe pneumonia with an AUC of 0.754 (95% CI (0.616~0.891)) and a cut-off value of 0.92 mg/mL with a sensitivity of 72.2% and specificity of 73.1%. The AUC of LDH combined with ApoA1 to predict death in patients with severe pneumonia was 0.873 (95% CI (0.779 ~0.967)), with a higher area under the line than the test alone, sensitivity of 85.14% and specificity of 82.83% (Figure 1).

### Analysis of risk factors for death from severe pneumonia

The effect of variables such as pneumonia staging, age, LDH and ApoA1 on death from severe pneumonia was sequentially assessed using one-way dichotomous logistic regression analysis, and variables with statistical significance (*P*<0.05) were included in the multi-factor dichotomous logistic regression analysis and regression models were developed. The results revealed that LDH > 289 U/mL and ApoA1 < 0.92 mg/mL would increase the risk of death from severe pneumonia, with statistically significant differences (OR=4.275, 0.548, *P*<0.05) (Table 6).

**Table 6:** Logistic regression analysis of risk factors for death from severe pneumonia.

Variablesm	Crude OR	95%CI	P value	Adjusted OR	95%CI	P value
SHAP	0.412	0.243~0.715	0.003	0.812	0.461~1.521	0.537
Age>70	0.347	0.186~0.637	0.001	1.176	0.726~1.871	0.465
LDH>289 U/mL	4.059	3.027~5.426	<0.001	4.275	2.758~6.652	<0.001
ApoA1<0.92 mg/mL	1.694	1.147~2.487	0.007	0.548	0.337~0.893	0.014

## Discussion

The pathogenesis of severe pneumonia is complex, and its exact pathogenesis is not yet clear. In essence, the systemic inflammatory response to severe pneumonia and consequently MODS is the result of a combination of excessive activation of the body's immune defense mechanisms leading to self-destruction and endocrine disruption due to increased energy expenditure of the body in a high catabolic state, not exclusively due to a single factor of direct damage by bacterial toxins [8] [9]. In a study looking at the survival curves of patients with major trauma [10], it was found that most patients survived the early stages of trauma but died in a secondary »ate death spurt«, which was thought to be related to the high catabolic state of critically ill patients. Severe pneumonia occurs early in the disease in a hypercatabolic state that leads to a cascade of new clinical problems, either leading to new organ failure or exacerbating existing organ failure. Patients will successively experience metabolic disorders such as decreased appetite, skeletal muscle proteolysis, increased gluconeogenesis, and insulin resistance in a short period of time, and the amount of protein consumption increases dramatically in a short period of time, making it difficult to maintain the basic energy needs of the body because the patient's own nutritional level decreases rapidly [11]. Therefore, finding indicators that can effectively assess a patient's condition can be a boon to the clinical management of SP.

Patients with SP can suffer from vital organ disorders triggered by pulmonary inflammation, among which myocardial injury is a common symptom in SP patients, and its occurrence correlates with myocardial ischemia caused by respiratory insufficiency, fever, and pathogenic bacterial infections in SP patients, while myocardial injury leads to an increase in the expression of cardiac enzymes in the blood, and the trend of changes in this biological index is closely related to exacerbation of the disease [12] [13]. LDH is an important indicator of myocardial injury and is highly reflective of the severity of myocardial injury [14]. The rise in LDH expression in the serum of patients with pneumonia may be due to a more intense inflammatory response in the lungs, accompanied by hypoxia, causing myocardial damage [15]. ApoA1 is an inflammatory factor with anti-inflammatory, antioxidant, and cholesterol-reversing effects, and is one of the components of HDL, which exerts its anti-inflammatory effects by inhibiting the expression of inflammatory cytokines [16] [17]. ApoA1 is closely correlated with the progression of pneumonia disease, and decreased expression may cause an increase in the expression of pro-inflammatory factors, which in turn decreases the ability of lung tissue to resist pathogens and further exacerbates the inflammatory response [18].

LDH and ApoA1 levels were found to correlate with age, with increased LDH levels and decreased ApoA1 levels in patients older than 70 years with severe pneumonia, which correlated with poorer immune function status and more severe muscle loss in older patients. In this study, patients with severe pneumonia had elevated LDH levels or reduced ApoA1 levels during hospitalization with acute exacerbation of chronic obstructive pulmonary disease or MODS, as both acute exacerbation of chronic obstructive pulmonary disease and MODS are severe wasting diseases, which, when combined with severe pneumonia, lead to an extremely serious inflammatory »waterfall effect« that severely impairs the immune function of the body [19] [20], and this blow is often fatal. Studies have shown that the combination of chronic obstructive pulmonary disease or MODS is an independent risk factor for death in patients with severe pneumonia [21]. Therefore, in the clinical treatment of severe pneumonia combined with acute exacerbation of chronic obstructive pulmonary disease, or severe pneumonia combined with multi-organ failure, in addition to active anti-infection, the patient should pay attention to the strength of nutrition and immune support.

The study also found increased LDH levels and decreased ApoA1 levels in patients with ICU stays >10 d and duration of mechanical ventilation >180 h. In strictly bedridden healthy adults, muscle strength decreases by 1% per day, while in the ICU, patients with severe pneumonia on mechanical ventilation and sedation and analgesia are generally in a state of complete or near-complete braking, with poorer status of various physiological functions, which severely affects the balance of myostatin synthesis and catabolism and makes them more susceptible to myocardial injury [22]. In addition, factors such as invasive manipulation and delirium states tend to aggravate the energy expenditure of the body [23]. ICU stays of more than 10 d are increasingly characterized by sustained catabolism [24], and sustained high catabolism leads to cachexia, severely impairs host immune function, causes severe muscle atrophy and ICU-related debilitation, and ultimately leads to prolonged hospitalization, poor prognosis, and even death in patients with severe pneumonia.

Currently, NLR, HCT, CRP, PCT, DD, PSI score, and APACHE II score are commonly used in China to predict the prognosis of patients with severe pneumonia [21] [22], and this study analyzed the above indicators and found that LDH and ApoA1 levels in the death group of patients with severe pneumonia were higher than those in the survival group, while the distribution levels of the remaining indicators did not differ significantly between the death and survival groups. In the prognostic assessment of patients with severe pneumonia, NLR, HCT, DD, PSI scores, and APACHE II scores may lead to a lag in clinical judgment of the severity of severe pneumonia and delay the timing of treatment. In contrast, CRP is an acute temporal response protein for various inflammatory diseases in the body. Although its sensitivity is high, it is subject to many interfering factors, and trauma, stress, postoperative and any infectious diseases can cause an increase in CRP, so its specificity is low, and its use to predict the prognosis of severe pneumonia can easily misjudge the disease. This may be related to the direct toxic effect of PCT on glomerular thylakoid cells and the reduced clearance of PCT by the kidney when renal function deteriorates. It may also be related to the delayed production and impaired release of PCT in elderly patients with severe pneumonia and the inability of a single time point PCT value to reflect the dynamic changes in lung inflammation, thus reducing its predictive efficacy [25], and thus its sensitivity and specificity for predicting the prognosis of severe pneumonia are not optimal. The AUC of LDH combined with ApoA1 for predicting death in patients with severe pneumonia was 0.873 (95% CI (0.779~0.967)), with a higher area under the line than the test alone, sensitivity of 85.14% and specificity of 82.83%, making it a prognostic indicator with a higher combined sensitivity and specificity compared to other indicators. The study suggests that LDH > 289 U/mL and ApoA1 < 0.92 mg/mL are independent risk factors for severe pneumonia. LDH and ApoA1 are clinically accessible, often repeatedly tested, quick and easy indicators, which are more ideal clinical markers of catabolic status in patients with severe pneumonia and can be easily promoted in primary hospitals. Therefore, the use of LDH and ApoA1 to predict the condition and prognosis of patients with severe pneumonia has some clinical value.

In conclusion, elevated LDH levels and reduced ApoA1 levels in patients with severe pneumonia are valuable in assessing patients' conditions and prognosis, and can provide assistance in the early assessment of patients' conditions and diagnosis and treatment. The study has some limitations. Firstly, the design method is a single-center retrospective study, which is influenced by various factors, the data collection is difficult, some data are missing, and there may be bias and unknown confounders. Secondly, the number of cases was limited and the sample size was not large enough. And there are more confounding factors to be excluded for the study index, and the extrapolation of its predictive efficacy is not good enough. Finally, the study analyzed the morbidity and mortality of critically ill patients during hospitalization and did not follow up on the post-discharge outcomes of surviving patients to allow for survival analysis. Therefore, large sample, multicenter, prospective studies are needed to confirm its clinical utility.

## Dodatak

### Conflict of interest statement

All the authors declare that they have no conflict of interest in this work.
